# Supplementary material for: Characterization of alcohol polygenic risk scores in the context of mental health outcomes: Within-individual and intergenerational analyses in the Avon Longitudinal Study of Parents and Children
Source: Drug Alcohol Depend. 2021 Apr 1;221:108654. doi: 10.1016/j.drugalcdep.2021.108654 (PMC8047864; doi:10.1016/j.drugalcdep.2021.108654)
Supplement: Supplementary file 1 [file mmc1.docx]

| **Supplementary information** |
| --- |
| **Supplementary Table 1. SNPs used to make alcohol PRS** |
| rs705687 |
| rs58107686 |
| rs12088813 |
| rs5024204 |
| rs10753661 |
| rs28680958 |
| rs823114 |
| rs77165542 |
| rs1260326 |
| rs2178197 |
| rs13383034 |
| rs1004787 |
| rs13032049 |
| rs828867 |
| rs11692435 |
| rs13024996 |
| rs72859280 |
| rs56337305 |
| rs13094887 |
| rs62250685 |
| rs74664784 |
| rs13066454 |
| rs9838144 |
| rs2011092 |
| rs60654199 |
| rs6787172 |
| rs3748034 |
| rs7682824 |
| rs11940694 |
| rs35538052 |
| rs4501255 |
| rs12499107 |
| rs144198753 |
| rs1154414 |
| rs1229984 |
| rs10028756 |
| rs561222871 |
| rs36052336 |
| rs2165670 |
| rs17029090 |
| rs79139602 |
| rs4699791 |
| rs13107325 |
| rs4690727 |
| rs10004020 |
| rs12651313 |
| rs4916723 |
| rs12655091 |
| rs55872084 |
| rs11739827 |
| rs10085696 |
| rs6460047 |
| rs10236149 |
| rs35034355 |
| rs6951574 |
| rs13250583 |
| rs1217091 |
| rs28601761 |
| rs55932213 |
| rs10978550 |
| rs7074871 |
| rs17665139 |
| rs7950166 |
| rs11030084 |
| rs56030824 |
| rs10750025 |
| rs1713676 |
| rs4938230 |
| rs682011 |
| rs12795042 |
| rs10876188 |
| rs3809162 |
| rs10506274 |
| rs4842786 |
| rs500321 |
| rs1123285 |
| rs2180870 |
| rs28929474 |
| rs11625650 |
| rs2472297 |
| rs12907323 |
| rs2764771 |
| rs17177078 |
| rs378421 |
| rs113443718 |
| rs62044525 |
| rs7185555 |
| rs79616692 |
| rs1104608 |
| rs4548913 |
| rs3803800 |
| rs2854334 |
| rs2532276 |
| rs10438820 |
| rs9950000 |
| rs4092465 |
| rs281379 |
| rs4815364 |
| rs9607814 |

| **Supplementary Table 2: Adjusted r^2^ values between own alcohol PRS and alcohol phenotypes** | | |
| --- | --- | --- |
| Mother | Beta | R^2^ |
| Alcohol amount | 0.041 | 1.6% |
| Binge drinking frequency (18 weeks) | 0.030 | 0.1% |
| Weekly units | 0.251 | 1.1% |
| Binge drinking frequency (32 weeks) | 0.036 | 0.5% |
| Child |  |  |
| Alcohol frequency | 0.024 | 2.8% |
| Audit risk score | 0.016 | 0.9% |
| Binge drinking frequency | 0.010 | 2.4% |
| Number of drinks to feel tipsy | -0.024 | 4.2% |
| Average drink total per day | -0.004 | 0.5% |
| Number of times had whole drink | 0.346 | 0.06% |
| AUDIT total score | 0.127 | 3.0% |

| **Supplementary Table 3: Associations between alcohol PRS and alcohol phenotypes for mothers and offspring** | | | | | | | |
| --- | --- | --- | --- | --- | --- | --- | --- |
| Exposure | Age | Effect Size | OR/beta | lowerCI | upperCI | pvalue | *n* |
| **Maternal outcomes** | | |  |  |  |  |  |
| Alcohol amount | 18 weeks gestation | beta | 0.041 | 0.02 | 0.06 | 1.01×10^-5^ | 7185 |
| Weekly units | 32 weeks gestation | beta | 0.251 | 0.14 | 0.36 | 1.70×10^-5^ | 4294 |
| Binge drinking frequency | 32 weeks gestation | beta | 0.036 | 0.02 | 0.06 | 2.37×10^-4^ | 5324 |
| Binge drinking frequency | 18 weeks gestation | beta | 0.030 | 0.01 | 0.05 | 0.001 | 7171 |
| Most consumed alcoholic drink: Wine | 18 weeks gestation | OR | 1.109 | 1.04 | 1.18 | 0.004 | 5199 |
| Reduced alcohol amount | 8 weeks gestation | OR | 1.077 | 1.02 | 1.14 | 0.011 | 6771 |
| Never drinker | 8 weeks gestation | OR | 0.925 | 0.87 | 0.98 | 0.017 | 6771 |
| Most consumed alcoholic drink: Beer/lager | 18 weeks gestation | OR | 1.101 | 1.02 | 1.19 | 0.021 | 3667 |
| No change in alcohol amount | 8 weeks gestation | OR | 0.930 | 0.85 | 1.02 | 0.101 | 6771 |
| Most consumed alcoholic drink: Other alcohol | 18 weeks gestation | OR | 1.065 | 0.96 | 1.18 | 0.212 | 3054 |
| Craved more alcohol | 8 weeks gestation | OR | 0.760 | 0.46 | 1.27 | 0.264 | 6771 |
| Stopped drinking alcohol | 8 weeks gestation | OR | 1.025 | 0.96 | 1.09 | 0.424 | 6771 |
| Most consumed alcoholic drink: Spirits | 18 weeks gestation | OR | 1.065 | 0.83 | 1.37 | 0.593 | 2582 |
| Most consumed alcoholic drink: Sherry/port | 18 weeks gestation | OR | 1.048 | 0.79 | 1.39 | 0.723 | 2540 |
| **Child outcomes** | |  |  |  |  |  |  |
| Alcohol frequency | 18 | beta | 0.024 | -0.01 | 0.05 | 0.116 | 2886 |
| AUDIT total score | 24 | beta | 0.127 | -0.03 | 0.29 | 0.121 | 2696 |
| AUDIT risk score | 18 | beta | 0.016 | -0.01 | 0.04 | 0.188 | 3008 |
| Number of times had whole alcoholic drink | 13 | beta | 0.346 | -0.18 | 0.87 | 0.198 | 1103 |
| Binge drinking frequency | 18 | beta | 0.010 | -0.04 | 0.06 | 0.675 | 2829 |
| Number of drinks to feel tipsy | 18 | beta | -0.025 | -0.15 | 0.10 | 0.698 | 2391 |
| Average drink total per day | 18 | beta | -0.004 | -0.05 | 0.04 | 0.859 | 2826 |
| **Intergenerational outcomes** | | | |  |  |  |  |
| AUDIT total score | 18 | beta | 0.184 | 0.02 | 0.35 | 0.028 | 2516 |
| Number of times had whole alcoholic drink | 18 | beta | 0.437 | -0.07 | 0.94 | 0.090 | 1012 |
| Average drink total per day | 18 | beta | -0.023 | -0.07 | 0.02 | 0.326 | 2647 |
| Alcohol frequency | 24 | beta | 0.012 | -0.02 | 0.04 | 0.440 | 2702 |
| Binge drinking frequency | 18 | beta | 0.013 | -0.03 | 0.06 | 0.594 | 2651 |
| AUDIT risk score | 18 | beta | 0.006 | -0.02 | 0.03 | 0.630 | 2812 |
| Number of drinks to feel tipsy | 18 | beta | -0.005 | -0.12 | 0.11 | 0.932 | 2246 |

| **Supplementary Table 4: Associations between maternal alcohol PRS and maternal mental health phenotypes** | | | | | | | |
| --- | --- | --- | --- | --- | --- | --- | --- |
| Phenotype | Age | Type | OR/beta | lowerCI | upperCI | pvalue | *n* |
| Depression | 32 weeks | OR | 1.097 | 1.02 | 1.18 | 0.022 | 6751 |
| Neuroticism | 18 weeks | beta | 0.164 | -0.02 | 0.35 | 0.078 | 6456 |
| Life events | 32 weeks | beta | 0.013 | 0.00 | 0.03 | 0.084 | 6936 |
| Education | 32 weeks | beta | 0.024 | 0.00 | 0.05 | 0.102 | 6956 |
| Smoked 1-3 months | 18 weeks | OR | 1.049 | 0.98 | 1.12 | 0.126 | 7237 |
| Depression | 18 weeks | OR | 1.063 | 0.98 | 1.15 | 0.131 | 6734 |
| Ever smoked | 8 weeks | OR | 0.958 | 0.90 | 1.02 | 0.152 | 6719 |
| Social class | 32 weeks | beta | -0.019 | -0.05 | 0.01 | 0.164 | 5854 |
| Reduced cigarettes | 8 weeks | OR | 1.051 | 0.98 | 1.13 | 0.174 | 6719 |
| Increased cigarettes | 8 weeks | OR | 1.421 | 0.79 | 2.54 | 0.213 | 6719 |
| Smoked cannabis 1-3 months | 18 weeks | OR | 1.104 | 0.94 | 1.30 | 0.214 | 6918 |
| Vomited in pregnancy | 18 weeks | OR | 0.969 | 0.92 | 1.02 | 0.225 | 6797 |
| Daily caffeine intake | 8 weeks | beta | 1.408 | -1.16 | 3.98 | 0.283 | 6769 |
| Hypersensitivity to rejection | 18 weeks | beta | 0.177 | -0.20 | 0.55 | 0.351 | 7169 |
| Sleep initiation | 32 weeks | beta | 0.008 | -0.01 | 0.03 | 0.417 | 6745 |
| No change in caffeine | 8 weeks | OR | 0.980 | 0.93 | 1.03 | 0.431 | 7269 |
| Image perception | 18 weeks | beta | 0.036 | -0.06 | 0.13 | 0.474 | 6701 |
| Increased caffeine | 8 weeks | OR | 0.971 | 0.89 | 1.07 | 0.510 | 7269 |
| Physical activity perception | 32 weeks | beta | 0.006 | -0.01 | 0.02 | 0.515 | 6716 |
| Craved more caffeine | 8 weeks | OR | 1.023 | 0.94 | 1.11 | 0.552 | 7269 |
| Ever drank caffeine | 8 weeks | OR | 0.987 | 0.94 | 1.04 | 0.585 | 7269 |
| Reduced caffeine | 8 weeks | OR | 0.990 | 0.94 | 1.04 | 0.675 | 7269 |
| No change in cigarettes | 8 weeks | OR | 1.017 | 0.90 | 1.15 | 0.783 | 6719 |
| Image perception change | 18 weeks | beta | -0.010 | -0.10 | 0.08 | 0.824 | 6551 |
| Stopped smoking | 8 weeks | OR | 1.010 | 0.91 | 1.12 | 0.830 | 6719 |
| Illicit drugs in pregnancy | 18 weeks | OR | 0.957 | 0.62 | 1.48 | 0.830 | 7147 |
| Reaction to becoming a parent | 18 weeks | beta | -0.002 | -0.02 | 0.02 | 0.831 | 7167 |
| Craved more cigarettes | 8 weeks | OR | 1.020 | 0.65 | 1.61 | 0.926 | 6719 |
| Average income | 33/47 months | beta | 0.000 | -0.03 | 0.03 | 0.983 | 5430 |

| **Supplementary Table 5: Associations between offspring alcohol PRS and offspring mental health phenotypes** | | | | | | | |
| --- | --- | --- | --- | --- | --- | --- | --- |
| Phenotype | Age | Type | OR/beta | lowerCI | upperCI | pvalue | *n* |
| **Children** |  |  |  |  |  |  |  |
| Handedness | 10 | OR | 0.922 | 0.84 | 1.01 | 0.071 | 5399 |
| Sleep duration | 7 | beta | -0.020 | -0.04 | 0.00 | 0.087 | 5445 |
| Sleep maintenance | 7 | OR | 0.954 | 0.90 | 1.02 | 0.139 | 5451 |
| Conduct disorder | 7 | beta | 0.003 | -0.02 | 0.02 | 0.767 | 5329 |
| Specific phobia | 10 | OR | 1.142 | 0.82 | 1.59 | 0.402 | 5473 |
| Life events | 7 | beta | 0.008 | -0.01 | 0.03 | 0.405 | 5496 |
| Daily caffeine intake | 8 | beta | 0.268 | -0.56 | 1.09 | 0.525 | 4589 |
| Oppositional defiant disorder | 7 | beta | 0.007 | -0.01 | 0.03 | 0.526 | 4859 |
| Hyperactivity symptoms | 7 | beta | -0.005 | -0.02 | 0.01 | 0.549 | 5222 |
| BMI | 7 | beta | -0.015 | -0.07 | 0.04 | 0.582 | 5799 |
| Total behavioural difficulties | 7 | beta | 0.023 | -0.10 | 0.15 | 0.709 | 5455 |
| Emotional symptoms score | 7 | beta | 0.006 | -0.04 | 0.05 | 0.787 | 5462 |
| Anxiety symptoms score | 8 | beta | -0.006 | -0.05 | 0.04 | 0.809 | 5358 |
| Depression | 10 | beta | 0.004 | -0.08 | 0.09 | 0.932 | 5434 |
| IQ | 8 | beta | -0.004 | -0.43 | 0.42 | 0.984 | 5290 |
| Sleep initiation | 7 | OR | 1.000 | 0.94 | 1.06 | 0.993 | 5479 |
|  |  |  |  |  |  |  |  |
| **Adolescents** | |  |  |  |  |  |  |
| Frequency smokes cannabis | 16 | beta | -0.072 | -0.15 | 0.01 | 0.078 | 1035 |
| Ever smoked | 14 | OR | 1.062 | 0.98 | 1.15 | 0.125 | 4145 |
| Lifetime cigarettes smoked | 18 | beta | 0.071 | -0.02 | 0.17 | 0.142 | 1144 |
| Conscientiousness | 13 | beta | -0.128 | -0.3 | 0.05 | 0.151 | 4162 |
| Conduct disorder | 16 | beta | 0.031 | -0.12 | 0.07 | 0.16 | 2871 |
| Ever smoked cannabis | 16 | OR | 0.949 | 0.88 | 1.03 | 0.182 | 3573 |
| Lifetime cigarettes smoked | 14 | OR | 1.099 | 0.95 | 1.27 | 0.193 | 1058 |
| Emotional stability | 13 | beta | -0.109 | -0.3 | 0.08 | 0.26 | 4224 |
| Psychosis negative symptoms | 16 | beta | -0.096 | -0.27 | 0.08 | 0.276 | 3513 |
| Number of cigarettes smoked daily | 23 | beta | -0.003 | -0.01 | 0 | 0.315 | 7841 |
| Education | 18 | OR | 0.955 | 0.87 | 1.05 | 0.324 | 2182 |
| Number of cigarettes smoked weekly | 23 | beta | 0.003 | 0 | 0.01 | 0.358 | 7841 |
| Daily caffeine intake | 13 | beta | 0.573 | -0.65 | 1.8 | 0.359 | 3405 |
| Eating disorder | 13 | OR | 0.8 | 0.47 | 1.35 | 0.375 | 4256 |
| Oppositional defiant disorder | 15 | beta | -0.011 | -0.04 | 0.02 | 0.42 | 2948 |
| Age of first cigarette | 18 | beta | -0.051 | -0.18 | 0.08 | 0.427 | 1131 |
| Exercise frequency | 14 | beta | 0.009 | -0.02 | 0.03 | 0.455 | 4270 |
| Extraversion | 13 | beta | 0.069 | -0.13 | 0.27 | 0.503 | 4354 |
| Depression | 18 | beta | -0.061 | -0.24 | 0.12 | 0.509 | 3212 |
| Depression symptom score | 18 | beta | 0.043 | -0.09 | 0.17 | 0.521 | 3303 |
| ADHD | 16 | beta | -0.013 | -0.05 | 0.28 | 0.526 | 2896 |
| Depression | 14 | beta | 0.039 | -0.09 | 0.17 | 0.554 | 4574 |
| Phobia symptom score | 18 | beta | 0.006 | -0.02 | 0.03 | 0.584 | 3293 |
| Eating disorder | 16 | OR | 1.078 | 0.78 | 1.5 | 0.627 | 3545 |
| Autism | 11 | OR | 0.934 | 0.69 | 1.27 | 0.637 | 5381 |
| Life events | 16 | beta | 0.004 | -0.01 | 0.02 | 0.642 | 3378 |
| Anxiety | 18 | beta | -0.006 | -0.03 | 0.02 | 0.645 | 3293 |
| Suicide attempt | 16 | OR | 0.97 | 0.83 | 1.14 | 0.684 | 3263 |
| Sleep maintenance | 15 | beta | 0.005 | -0.02 | 0.03 | 0.685 | 3419 |
| Total behavioural difficulties | 17 | beta | -0.028 | -0.18 | 0.12 | 0.709 | 4055 |
| Lifetime cigarettes smoked | 23 | beta | 0.007 | -0.03 | 0.04 | 0.712 | 7841 |
| Intellectual ability | 13 | beta | -0.026 | -0.19 | 0.14 | 0.759 | 4263 |
| IQ | 15 | beta | -0.058 | -0.48 | 0.36 | 0.786 | 3721 |
| Sleep duration | 16 | beta | 0.004 | -0.03 | 0.04 | 0.803 | 3727 |
| Psychosis positive symptoms | 12 | beta | -0.002 | -0.02 | 0.01 | 0.804 | 4974 |
| Ever smoked | 18 | OR | 0.991 | 0.91 | 1.08 | 0.831 | 2402 |
| Agreeableness | 13 | beta | 0.015 | -0.13 | 0.16 | 0.836 | 4279 |
| Age of first cigarette | 14 | beta | 0.002 | -0.04 | 0.04 | 0.919 | 1064 |
| BMI | 17 | beta | -0.006 | -0.13 | 0.12 | 0.93 | 3606 |
| Ever smoked | 23 | OR | 0.997 | 0.91 | 1.09 | 0.94 | 2792 |
| Sleep initiation | 15 | beta | 0.017 | -0.47 | 0.5 | 0.945 | 3627 |
| Education | 18 | OR | 1.005 | 0.79 | 1.28 | 0.965 | 2360 |
| Psychosis positive symptoms | 18 | beta | 0 | -0.02 | 0.02 | 0.968 | 3403 |
| Psychosis positive symptoms | 17 | beta | 0.001 | -0.06 | 0.06 | 0.981 | 4073 |
| PTSD | 15 | beta | 0 | -0.01 | 0.01 | 0.983 | 4009 |

| **Supplementary Table 6: Associations between maternal alcohol PRS and offspring mental health phenotype (intergenerational)** | | | | | | | |
| --- | --- | --- | --- | --- | --- | --- | --- |
| Phenotype | Age | Type | OR/beta | lowerCI | upperCI | pvalue | *n* |
| Intellectual ability | 13 | beta | -0.209 | -0.38 | -0.04 | 0.016 | 3956 |
| Daily caffeine intake | 8 | beta | 0.774 | -0.04 | 1.59 | 0.064 | 4067 |
| Exercise frequency | 14 | beta | 0.022 | 0.00 | 0.05 | 0.086 | 3969 |
| Conscientiousness | 13 | beta | -0.158 | -0.34 | 0.02 | 0.090 | 3863 |
| Depression | 10 | beta | 0.073 | -0.02 | 0.16 | 0.112 | 4885 |
| Education | 18 | OR | 0.927 | 0.84 | 1.02 | 0.122 | 2038 |
| Depression | 14 | beta | 0.100 | -0.03 | 0.23 | 0.142 | 4250 |
| Ever smoked | 23 | OR | 1.065 | 0.98 | 1.16 | 0.145 | 2610 |
| IQ | 15 | beta | 0.320 | -0.13 | 0.77 | 0.161 | 3450 |
| BMI | 17 | beta | -0.087 | -0.22 | 0.05 | 0.214 | 3353 |
| Total behavioural difficulties | 7 | beta | 0.079 | -0.05 | 0.21 | 0.217 | 5135 |
| BMI | 7 | beta | -0.035 | -0.09 | 0.02 | 0.223 | 5032 |
| Age of first cigarette | 14 | beta | -0.026 | -0.07 | 0.02 | 0.246 | 970 |
| Ever smoked | 14 | OR | 1.044 | 0.96 | 1.13 | 0.271 | 3876 |
| Frequency smokes cannabis | 16 | beta | -0.041 | -0.12 | 0.03 | 0.285 | 960 |
| Education | 18 | OR | 1.132 | 0.89 | 1.44 | 0.286 | 2204 |
| Extraversion | 13 | beta | 0.109 | -0.10 | 0.32 | 0.309 | 4046 |
| Lifetime cigarettes smoked | 14 | OR | 1.078 | 0.92 | 1.26 | 0.316 | 965 |
| Sleep initiation | 15 | beta | -0.266 | -0.79 | 0.25 | 0.316 | 3355 |
| Agreeableness | 13 | beta | -0.076 | -0.23 | 0.07 | 0.323 | 3983 |
| Anxiety | 18 | beta | -0.013 | -0.04 | 0.01 | 0.331 | 3051 |
| Sleep maintenance | 7 | OR | 0.971 | 0.91 | 1.04 | 0.348 | 5129 |
| Conduct disorder | 7 | beta | 0.009 | -0.011 | 0.029 | 0.378 | 5014 |
| Hyperactivity symptoms | 7 | beta | 0.008 | -0.01 | 0.03 | 0.385 | 4918 |
| Psychosis positive symptoms | 18 | beta | -0.007 | -0.02 | 0.01 | 0.414 | 3180 |
| Conduct disorder | 16 | beta | -0.02 | -0.03 | 0.06 | 0.437 | 3538 |
| Ever smoked | 18 | OR | 1.034 | 0.94 | 1.13 | 0.455 | 2239 |
| Lifetime cigarettes smoked | 18 | beta | -0.036 | -0.13 | 0.06 | 0.460 | 1041 |
| Sleep maintenance | 15 | beta | 0.010 | -0.02 | 0.04 | 0.461 | 3179 |
| Age of first cigarette | 18 | beta | -0.050 | -0.18 | 0.08 | 0.465 | 1038 |
| Lifetime cigarettes smoked | 23 | beta | 0.013 | -0.02 | 0.05 | 0.476 | 7727 |
| Depression | 18 | beta | -0.068 | -0.26 | 0.12 | 0.483 | 3015 |
| Eating disorder | 16 | OR | 1.098 | 0.81 | 1.49 | 0.518 | 3310 |
| Psychosis positive symptoms | 17 | beta | 0.018 | -0.04 | 0.08 | 0.542 | 3765 |
| Autism | 11 | OR | 1.082 | 0.79 | 1.48 | 0.593 | 4910 |
| Ever smoked cannabis | 16 | OR | 1.021 | 0.94 | 1.11 | 0.617 | 3335 |
| Psychosis negative symptoms | 16 | beta | 0.045 | -0.14 | 0.23 | 0.626 | 3273 |
| Sleep initiation | 7 | OR | 1.014 | 0.95 | 1.08 | 0.645 | 5152 |
| Total behavioural difficulties | 17 | beta | 0.033 | -0.13 | 0.19 | 0.684 | 3748 |
| Number of cigarettes smoked weekly | 23 | beta | 0.001 | -0.01 | 0.01 | 0.726 | 7727 |
| Eating disorder | 13 | OR | 1.080 | 0.67 | 1.73 | 0.731 | 3956 |
| Sleep duration | 7 | beta | -0.004 | -0.03 | 0.02 | 0.748 | 5129 |
| Phobia symptom score | 18 | beta | 0.004 | -0.02 | 0.03 | 0.750 | 3051 |
| Life events | 16 | beta | -0.003 | -0.02 | 0.02 | 0.762 | 3148 |
| Specific phobia | 10 | OR | 1.036 | 0.80 | 1.34 | 0.766 | 5102 |
| IQ | 8 | beta | 0.069 | -0.40 | 0.53 | 0.771 | 4675 |
| Handedness | 10 | OR | 1.012 | 0.92 | 1.11 | 0.778 | 4849 |
| Number of cigarettes smoked daily | 23 | beta | -0.001 | -0.01 | 0.00 | 0.789 | 7727 |
| Psychosis positive symptoms | 12 | beta | -0.002 | -0.02 | 0.01 | 0.809 | 4568 |
| Oppositional defiant disorder | 7 | beta | -0.003 | -0.02 | 0.02 | 0.813 | 4492 |
| Sleep duration | 16 | beta | -0.004 | -0.04 | 0.03 | 0.817 | 3456 |
| Emotional stability | 13 | beta | 0.022 | -0.18 | 0.23 | 0.832 | 3901 |
| Anxiety symptoms score | 8 | beta | 0.004 | -0.04 | 0.05 | 0.860 | 4995 |
| Daily caffeine intake | 13 | beta | -0.099 | -1.28 | 1.08 | 0.870 | 3157 |
| Suicide attempt | 16 | OR | 1.012 | 0.86 | 1.19 | 0.875 | 3076 |
| Depression symptom score | 18 | beta | 0.009 | -0.13 | 0.15 | 0.894 | 3059 |
| Emotional symptoms score | 7 | beta | 0.003 | -0.04 | 0.05 | 0.904 | 5141 |
| ADHD | 16 | beta | -0.002 | -0.04 | 0.04 | 0.908 | 3566 |
| PTSD | 15 | beta | 0.000 | -0.01 | 0.01 | 0.957 | 3722 |
| Life events | 7 | beta | 0.000 | -0.02 | 0.02 | 0.989 | 5169 |
| Oppositional defiant disorder | 15 | beta | 0.000 | -0.03 | 0.03 | 0.999 | 2759 |

| **Supplementary Table 7: Permutation analyses of maternal pregnancy phenotypes and maternal PRS for alcohol consumption** | | | | |
| --- | --- | --- | --- | --- |
| Phenotype | Tobs | prop | lowerCI | upperCI |
| Depression 32 weeks | 0.092430986 | 0.016000001 | 0.009172319 | 0.025853248 |
| Neuroticism | 0.16413787 | 0.123000003 | 0.103276908 | 0.144972235 |
| Depression 18 weeks | 0.060914848 | 0.126000002 | 0.106056832 | 0.14817372 |
| Smoked 1-3 months | 0.047895133 | 0.128999993 | 0.10884057 | 0.151371419 |
| Never smoked | -0.042689145 | 0.129999995 | 0.109769315 | 0.152436495 |
| Life events | 0.012600807 | 0.158000007 | 0.135926515 | 0.182107598 |
| Reduced cigarettes | 0.049414 | 0.166 | 0.143449 | 0.190537 |
| Education | 0.023924 | 0.191 | 0.167075 | 0.216759 |
| Smoked cannabis 1-3 months | 0.098661 | 0.222 | 0.196591 | 0.249057 |
| Increased cigarettes | 0.351009 | 0.242 | 0.215746 | 0.269782 |
| Social class | -0.01878 | 0.267 | 0.2398 | 0.295579 |
| Vomited in pregnancy | -0.03171 | 0.279 | 0.251386 | 0.307921 |
| Daily caffeine intake | 1.407763 | 0.35 | 0.320416 | 0.38047 |
| No change in caffeine | -0.02003 | 0.374 | 0.343919 | 0.404825 |
| Hypersensitivity to rejection | 0.177089 | 0.419 | 0.388197 | 0.450282 |
| Sleep initiation | 0.008048 | 0.496 | 0.464562 | 0.527462 |
| Increased caffeine | -0.02893 | 0.514 | 0.48252 | 0.545397 |
| Image perception | 0.035877 | 0.527 | 0.495515 | 0.558326 |
| Craved more caffeine | 0.022349 | 0.564 | 0.532615 | 0.595008 |
| Physical activity perception | 0.005978 | 0.586 | 0.554756 | 0.616736 |
| Ever drank caffeine | -0.01337 | 0.606 | 0.574939 | 0.636435 |
| Reduced caffeine | -0.01024 | 0.663 | 0.632756 | 0.69228 |
| No change in cigarettes | 0.016403 | 0.816 | 0.790566 | 0.839558 |
| Illicit drugs in pregnancy | -0.04401 | 0.819 | 0.793709 | 0.842396 |
| Reaction to becoming a parent | -0.00236 | 0.839 | 0.814729 | 0.861255 |
| Image perception change | -0.00991 | 0.84 | 0.815783 | 0.862195 |
| Stopped smoking | 0.010352 | 0.853 | 0.829516 | 0.874383 |
| Craved more cigarettes | 0.019798 | 0.909 | 0.889449 | 0.926101 |
| Average income | -0.0003 | 0.985 | 0.97538 | 0.991581 |

| **Supplementary Table 8: Permutation analyses of child phenotypes and child PRS for alcohol consumption** | | | | |
| --- | --- | --- | --- | --- |
| Phenotype | Tobs | prop | lowerCI | upperCI |
| Sleep duration | -0.01964 | 0.223 | 0.197547 | 0.250095 |
| Handedness | -0.08106 | 0.229 | 0.203286 | 0.25632 |
| Sleep maintenance age 7 | -0.0466 | 0.243 | 0.216706 | 0.270816 |
| Frequency smokes cannabis | -0.07175 | 0.312 | 0.283373 | 0.341738 |
| Ever smoked age 23 | 0.059983 | 0.317 | 0.288235 | 0.346847 |
| Conscientiousness | -0.12798 | 0.393 | 0.362581 | 0.424051 |
| Lifetime cigarettes smoked age 14 | 0.094084 | 0.409 | 0.378334 | 0.440203 |
| Ever smoked cannabis | -0.05236 | 0.431 | 0.400049 | 0.462359 |
| Specific phobia | 0.132772 | 0.488 | 0.456593 | 0.519477 |
| Lifetime cigarettes smoked age 18 | 0.070771 | 0.503 | 0.471541 | 0.534441 |
| Eating disorder age 13 | -0.22319 | 0.522 | 0.490588 | 0.553814 |
| Daily caffeine intake age 13 | 0.57314 | 0.527 | 0.495515 | 0.558326 |
| Emotional stability | -0.10867 | 0.529 | 0.497516 | 0.560313 |
| Life events age 7 | 0.007643 | 0.548 | 0.51655 | 0.579166 |
| Psychosis negative symptoms age 16 | -0.0957 | 0.556 | 0.524578 | 0.587091 |
| Oppositional defiant disorder age 15 | -0.01115 | 0.564 | 0.532615 | 0.595008 |
| ADHD | -0.01920 | 0.545 | 0.513542 | 0.576192 |
| Daily caffeine intake age 8 | 0.2678 | 0.624 | 0.593149 | 0.654119 |
| Exercise frequency | 0.009444 | 0.643 | 0.612418 | 0.672737 |
| Oppositional defiant disorder age 7 | 0.006577 | 0.655 | 0.624614 | 0.68447 |
| Hyperactivity symptoms | -0.00523 | 0.683 | 0.653153 | 0.711765 |
| Extraversion | 0.06909 | 0.688 | 0.658262 | 0.716627 |
| Depression age 18 | -0.06117 | 0.688 | 0.658262 | 0.716627 |
| Education (GCSE D-G) | -0.04583 | 0.693 | 0.663374 | 0.721485 |
| BMI age 7 | -0.01497 | 0.708 | 0.678736 | 0.736034 |
| Age of first cigarette | -0.05119 | 0.725 | 0.69619 | 0.752479 |
| Autism | -0.06842 | 0.731 | 0.702362 | 0.758271 |
| Depression age 14 | 0.038703 | 0.737 | 0.708541 | 0.764057 |
| Depression symptom score | 0.042785 | 0.751 | 0.722983 | 0.777531 |
| Eating disorder age 16 | 0.075539 | 0.759 | 0.731252 | 0.785214 |
| Phobia symptom score | 0.006163 | 0.761 | 0.733321 | 0.787133 |
| Anxiety | -0.00605 | 0.783 | 0.756138 | 0.808185 |
| Number of cigarettes smoked daily | -0.0026 | 0.795 | 0.768627 | 0.819623 |
| Conduct disorder age 16 | 0.17025 | 0.619 | 0.588086 | 0.649211 |
| Life events age 16 | 0.004338 | 0.807 | 0.78115 | 0.831028 |
| Total behavioural difficulties age 7 | 0.023378 | 0.807 | 0.78115 | 0.831028 |
| Number of cigarettes smoked weekly | 0.002832 | 0.809 | 0.783241 | 0.832925 |
| Total behavioural difficulties age 17 | -0.02808 | 0.814 | 0.788472 | 0.837664 |
| Emotional symptoms score | 0.005791 | 0.829 | 0.804205 | 0.85184 |
| Conduct disorder age 7 | 0.002783 | 0.83 | 0.805256 | 0.852783 |
| Suicide attempt | -0.03096 | 0.841 | 0.816838 | 0.863134 |
| Sleep maintenance age 15 | 0.005252 | 0.844 | 0.820003 | 0.865951 |
| Intellectual ability | -0.02634 | 0.859 | 0.835873 | 0.879988 |
| Anxiety symptoms score | -0.00575 | 0.873 | 0.85076 | 0.893016 |
| IQ age 15 | -0.05847 | 0.876 | 0.85396 | 0.895797 |
| Psychosis positive symptoms | -0.00174 | 0.884 | 0.862513 | 0.903194 |
| Sleep duration | 0.003974 | 0.905 | 0.885115 | 0.92246 |
| Agreeableness | 0.015139 | 0.918 | 0.899237 | 0.934254 |
| Lifetime cigarettes smoked age 23 | 0.006595 | 0.921 | 0.902513 | 0.936959 |
| Ever smoked | -0.00911 | 0.929 | 0.911284 | 0.944135 |
| Depression age 10 | 0.003713 | 0.937 | 0.920112 | 0.951253 |
| Age of first cigarette | 0.002186 | 0.95 | 0.93461 | 0.962665 |
| Ever smoked age 18 | -0.00304 | 0.954 | 0.939116 | 0.966129 |
| BMI age 17 | -0.00573 | 0.955 | 0.940247 | 0.96699 |
| Sleep initiation age 15 | 0.017167 | 0.959 | 0.944788 | 0.97042 |
| Education (GCSE A-C) | 0.004978 | 0.973 | 0.960919 | 0.982115 |
| Psychosis positive symptoms age 18 | 0.000326 | 0.985 | 0.97538 | 0.991581 |
| PTSD | -0.00015 | 0.987 | 0.977872 | 0.99306 |
| IQ age 7 | -0.00425 | 0.988 | 0.979132 | 0.993784 |
| Psychosis positive symptoms | 0.000681 | 0.99 | 0.981687 | 0.995194 |
| Sleep initiation age 7 | -0.00027 | 0.996 | 0.98979 | 0.998909 |

| **Supplementary Table 9: Permutation analyses of intergenerational analyses. Child phenotypes and maternal PRS for alcohol consumption** | | | | |
| --- | --- | --- | --- | --- |
| Phenotype | Tobs | prop | lowerCI | upperCI |
| Daily caffeine intake age 8 | 0.777552 | 0.14 | 0.119078 | 0.163066 |
| Intellectual ability | -0.21082 | 0.154 | 0.132173 | 0.177885 |
| Ever smoked age 23 | 0.062897 | 0.167 | 0.14439 | 0.191589 |
| ADHD | 0.0408546 | 0.237 | 0.2109495 | 0.2646085 |
| Depression age 10 | 0.071943 | 0.251 | 0.224392 | 0.279083 |
| Conscientiousness | -0.15893 | 0.283 | 0.255254 | 0.31203 |
| Exercise frequency | 0.020345 | 0.307 | 0.278515 | 0.336626 |
| Depression age 14 | 0.107127 | 0.39 | 0.359631 | 0.421018 |
| BMI age 7 | -0.03377 | 0.392 | 0.361598 | 0.42304 |
| Total behavioural difficulties age 7 | 0.071877 | 0.412 | 0.381292 | 0.443228 |
| IQ age 15 | 0.314028 | 0.427 | 0.396096 | 0.458335 |
| Sleep maintenance age 7 | -0.02955 | 0.456 | 0.424799 | 0.487461 |
| Ever smoked | 0.043449 | 0.467 | 0.435714 | 0.498481 |
| Age of first cigarette | -0.02602 | 0.471 | 0.439687 | 0.502484 |
| Education (GCSE D-G) | -0.07612 | 0.48 | 0.448633 | 0.511485 |
| BMI age 17 | -0.08631 | 0.497 | 0.465559 | 0.528459 |
| Lifetime cigarettes smoked age 14 | 0.075523 | 0.527 | 0.495515 | 0.558326 |
| Extraversion | 0.111626 | 0.531 | 0.499517 | 0.5623 |
| Frequency smokes cannabis | -0.04767 | 0.556 | 0.524578 | 0.587091 |
| Conduct disorder age 16 | -0.002527 | 0.925 | 0.906892 | 0.940553 |
| Hyperactivity symptoms | 0.006829 | 0.57 | 0.538647 | 0.60094 |
| Sleep initiation age 15 | -0.25496 | 0.582 | 0.550726 | 0.61279 |
| Agreeableness | -0.07116 | 0.589 | 0.55778 | 0.619694 |
| Psychosis positive symptoms | 0.027886 | 0.589 | 0.55778 | 0.619694 |
| Education (GCSE A-C) | 0.123912 | 0.595 | 0.563832 | 0.625607 |
| Sleep maintenance age 15 | 0.011637 | 0.645 | 0.614449 | 0.674694 |
| Psychosis positive symptoms age 18 | -0.00647 | 0.66 | 0.629702 | 0.689353 |
| Anxiety | -0.01192 | 0.664 | 0.633775 | 0.693256 |
| Autism | 0.078919 | 0.696 | 0.666444 | 0.724397 |
| Depression age 18 | -0.05644 | 0.72 | 0.691051 | 0.747647 |
| Eating disorder age 16 | 0.093223 | 0.723 | 0.694134 | 0.750547 |
| Lifetime cigarettes smoked age 18 | -0.03711 | 0.734 | 0.705451 | 0.761164 |
| Sleep initiation age 7 | 0.013582 | 0.741 | 0.712663 | 0.76791 |
| Age of first cigarette | -0.04388 | 0.741 | 0.712663 | 0.76791 |
| Ever smoked age 18 | 0.033043 | 0.746 | 0.717821 | 0.772723 |
| Ever smoked cannabis | 0.020343 | 0.75 | 0.72195 | 0.77657 |
| Total behavioural difficulties age 17 | 0.034087 | 0.783 | 0.756138 | 0.808185 |
| Psychosis negative symptoms age 16 | 0.047366 | 0.786 | 0.759257 | 0.811047 |
| Oppositional defiant disorder age 7 | -0.00339 | 0.804 | 0.778016 | 0.82818 |
| Phobia symptom score | 0.00484 | 0.817 | 0.791613 | 0.840504 |
| Lifetime cigarettes smoked age 23 | 0.012947 | 0.819 | 0.793709 | 0.842396 |
| Eating disorder age 13 | 0.076669 | 0.825956 | 0.800927 | 0.849036 |
| Handedness | 0.012338 | 0.844 | 0.820003 | 0.865951 |
| Life events age 16 | -0.00312 | 0.846 | 0.822115 | 0.867827 |
| IQ age 7 | 0.068031 | 0.855 | 0.831634 | 0.876253 |
| Specific phobia | 0.035553 | 0.855 | 0.831634 | 0.876253 |
| Sleep duration | -0.00311 | 0.856 | 0.832693 | 0.877187 |
| Depression symptom score | 0.018364 | 0.878 | 0.856096 | 0.897649 |
| Emotional symptoms score | 0.004412 | 0.885 | 0.863584 | 0.904117 |
| Sleep duration | -0.00408 | 0.886 | 0.864656 | 0.905039 |
| Psychosis positive symptoms | -0.00163 | 0.886 | 0.864656 | 0.905039 |
| Anxiety symptoms score | 0.00454 | 0.887 | 0.865728 | 0.90596 |
| Conduct disorder age 7 | 0.008295 | 0.558 | 0.526587 | 0.589071 |
| Daily caffeine intake age 13 | -0.0987 | 0.905 | 0.885115 | 0.92246 |
| Number of cigarettes smoked weekly | 0.001097 | 0.921 | 0.902513 | 0.936959 |
| Suicide attempt | 0.01188 | 0.931 | 0.913485 | 0.945921 |
| Emotional stability | 0.012627 | 0.948 | 0.932365 | 0.960923 |
| Number of cigarettes smoked daily | -0.00066 | 0.956 | 0.94138 | 0.967851 |
| Life events age 7 | 0.000276 | 0.981 | 0.970488 | 0.988523 |
| Oppositional defiant disorder age 15 | 0.000314 | 0.988 | 0.979132 | 0.993784 |
| PTSD | 0.000116 | 0.996 | 0.98979 | 0.998909 |

| **Supplementary Table 10: Associations between maternal alcohol PRS and maternal mental health phenotype (intergenerational) excluding ALSPAC and 23andMe from summary statistics used to make PRS** | | | | | | |
| --- | --- | --- | --- | --- | --- | --- |
| Phenotype | OR/beta | OR/beta | lowerCI | upperCI | pvalue | *n* |
| Alcohol amt 32 weeks gest | beta | 0.245259196 | 0.128921449 | 0.361596942 | 3.64754E-05 | 4294 |
| Alcohol amt 18 weeks gest | beta | 0.037898879 | 0.019695647 | 0.056102112 | 4.52692E-05 | 7185 |
| Alcohol binge 32 weeks gest | beta | 0.032506377 | 0.013237665 | 0.05177509 | 0.000948536 | 5324 |
| Alcohol binge 18 weeks gest | beta | 0.028417487 | 0.010974826 | 0.045860149 | 0.001410693 | 7171 |
| Most consumed alcoholic drink: Wine | OR | 1.10274601 | 1.036457419 | 1.173274279 | 0.004918854 | 5199 |
| Reduced alcohol amount | OR | 1.074244022 | 1.01761055 | 1.134029388 | 0.013802237 | 6771 |
| Never drinker | OR | 0.932498693 | 0.877284527 | 0.99118793 | 0.028184464 | 6771 |
| Depression 32 weeks gest | OR | 1.087236404 | 1.006166816 | 1.174837947 | 0.03660138 | 6751 |
| Most consumed alcoholic drink: Beer/lager | OR | 1.086734056 | 1.004215717 | 1.17603302 | 0.040571567 | 3667 |
| anxiety | beta | 0.18732512 | 0.004383269 | 0.370266974 | 0.044759244 | 6456 |
| Depression 18 weeks gest | OR | 1.072107553 | 0.987282634 | 1.164220333 | 0.090545967 | 6734 |
| Life events | beta | 0.011853102 | -0.00238861 | 0.026094811 | 0.102824911 | 6936 |
| No change in alcohol amount | OR | 0.931428671 | 0.851287365 | 1.019114614 | 0.111040108 | 6771 |
| Smoked 1-3 months pregnancy | OR | 1.048656821 | 0.984089434 | 1.117460728 | 0.129281476 | 7237 |
| Increased cigarettes | OR | 1.542038798 | 0.859735727 | 2.765830755 | 0.132238001 | 6719 |
| Smoked cannabis 1-3 months | OR | 1.132902145 | 0.956114829 | 1.342377782 | 0.135028169 | 6918 |
| Reduced cigarettes | OR | 1.055706501 | 0.97968781 | 1.137623906 | 0.139974847 | 6719 |
| Education | beta | 0.020861758 | -0.00790263 | 0.049626149 | 0.155147299 | 6956 |
| Social class | beta | -0.0180929 | -0.04433445 | 0.008148652 | 0.176547095 | 5854 |
| Ever smoked | OR | 0.962098658 | 0.905427754 | 1.022316575 | 0.190756038 | 6719 |
| Craved more alcohol | OR | 0.712484896 | 0.410525173 | 1.236549616 | 0.205159605 | 6771 |
| Most consumed alcoholic drink: Other alcohol | OR | 1.062448382 | 0.956962764 | 1.179561734 | 0.230857834 | 3054 |
| No change in caffeine | OR | 0.96961391 | 0.918973327 | 1.023044944 | 0.23393786 | 7269 |
| Daily caffeine intake | beta | 1.534430385 | -1.04455209 | 4.113412857 | 0.24351868 | 6769 |
| Vomited in pregnancy | OR | 0.970756829 | 0.919800222 | 1.024536371 | 0.253556818 | 6797 |
| Increased caffeine | OR | 0.965666473 | 0.88009733 | 1.059555173 | 0.427989423 | 7269 |
| Physical activity perception | beta | 0.006495113 | -0.01154378 | 0.024534004 | 0.48031503 | 6716 |
| Image perception | beta | 0.034728643 | -0.06375986 | 0.133217156 | 0.489438593 | 6701 |
| Stopped drinking alcohol | OR | 1.019941449 | 0.956358612 | 1.087751389 | 0.516550601 | 6771 |
| Craved more caffeine | OR | 1.02357173 | 0.945066094 | 1.108598828 | 0.536635041 | 7269 |
| Sleep initiation | beta | 0.006065135 | -0.01410273 | 0.026233004 | 0.555517375 | 5886 |
| Neuroticism | beta | 0.100717373 | -0.27220452 | 0.47363928 | 0.596524 | 7169 |
| Most consumed alcoholic drink: Sherry/port | OR | 0.941318631 | 0.71969229 | 1.2311939 | 0.632416427 | 2540 |
| Craved more cigarettes | OR | 0.923555851 | 0.595407486 | 1.432557464 | 0.699988961 | 6719 |
| Most consumed alcoholic drink: Spirits | OR | 1.045693874 | 0.81685704 | 1.338637829 | 0.700364709 | 2582 |
| Hypersensitivity to rejection | beta | -0.00406663 | -0.02555902 | 0.017425772 | 0.710714042 | 7167 |
| Average income | beta | 0.005071924 | -0.02275893 | 0.032902773 | 0.720906734 | 5430 |
| Sleep duration | beta | 0.003438402 | -0.0160403 | 0.022917101 | 0.729325533 | 6745 |
| Ever drank caffeine | OR | 0.992229223 | 0.941999495 | 1.045137286 | 0.749163747 | 7269 |
| Reduced caffeine | OR | 0.994616032 | 0.944285631 | 1.047628999 | 0.824615002 | 7269 |
| Image perception change | beta | -0.00926121 | -0.09696353 | 0.078441113 | 0.836010814 | 6551 |
| Illicit drugs in pregnancy | OR | 0.974678934 | 0.633433461 | 1.499761343 | 0.898978114 | 7147 |
| No change in cigarettes | OR | 1.004932404 | 0.886085153 | 1.139720321 | 0.933526158 | 6719 |
| Stopped smoking | OR | 1.001404405 | 0.90408051 | 1.109205127 | 0.976632714 | 6719 |

| **Supplementary Table 11: Associations between offspring alcohol PRS and offspring mental health phenotype (intergenerational) excluding ALSPAC and 23andMe from summary statistics used to make PRS** | | | | | | |
| --- | --- | --- | --- | --- | --- | --- |
| Phenotype | Type | OR/beta | lowerCI | upperCI | pvalue | n |
| handedness | OR | 0.913937 | 0.835492 | 0.999748 | 0.049448 | 5399 |
| cannabisfreq | Beta | -0.06447 | -0.14343 | 0.014487 | 0.10941 | 1035 |
| conscientious13 | Beta | -0.13874 | -0.31523 | 0.03775 | 0.123346 | 4162 |
| kq260_7 | Beta | -0.01682 | -0.03929 | 0.005653 | 0.14237 | 5445 |
| sleep7_maint_bi | OR | 0.9552 | 0.895708 | 1.018643 | 0.147586 | 5451 |
| auditrisk18 | Beta | 0.017179 | -0.00613 | 0.040489 | 0.148528 | 3008 |
| totalcigs18 | Beta | 0.068874 | -0.02609 | 0.163839 | 0.155013 | 1144 |
| audittotal18 | Beta | 0.121779 | -0.05064 | 0.2942 | 0.166198 | 3008 |
| alcfreq18 | Beta | 0.021105 | -0.00897 | 0.051181 | 0.168949 | 2886 |
| audit24tot | Beta | 0.111923 | -0.04865 | 0.272501 | 0.171827 | 2696 |
| psychosis16_neg | Beta | -0.10911 | -0.28212 | 0.0639 | 0.21636 | 3513 |
| exercise14 | Beta | 0.015781 | -0.00955 | 0.041112 | 0.222006 | 4270 |
| timesdrank13 | Beta | 0.32148 | -0.20713 | 0.850091 | 0.233013 | 1103 |
| cannabis | OR | 0.956197 | 0.882382 | 1.036188 | 0.249886 | 3573 |
| CD_16_total_rev | Beta | 0.021553 | -0.01573 | 0.058834 | 0.257109 | 3836 |
| gcse_DG | OR | 0.950235 | 0.863213 | 1.04603 | 0.271597 | 2182 |
| norm_ADHD_16_total_rev | Beta | -0.01861 | -0.054 | 0.016787 | 0.302768 | 3854 |
| norm_YPC1371 | Beta | 0.003036 | -0.00292 | 0.008989 | 0.317381 | 7841 |
| extraversion13 | Beta | 0.098882 | -0.10567 | 0.303438 | 0.343333 | 4354 |
| eatingdis13 | OR | 0.791404 | 0.472417 | 1.325778 | 0.345171 | 4256 |
| smoked18 | Beta | -0.0602 | -0.18551 | 0.065103 | 0.346042 | 1131 |
| Gr_ODD_15 | Beta | -0.01282 | -0.03984 | 0.014206 | 0.352478 | 2948 |
| alcbinge18 | Beta | 0.021651 | -0.02555 | 0.068849 | 0.368493 | 2829 |
| caff_13_MS | Beta | 0.553885 | -0.66311 | 1.770875 | 0.372268 | 3405 |
| Gr_HYP_7 | Beta | -0.00726 | -0.02437 | 0.009853 | 0.40573 | 5222 |
| cisranx18 | Beta | -0.00978 | -0.03522 | 0.015655 | 0.450859 | 3293 |
| eatingdis16 | OR | 1.117565 | 0.817209 | 1.528313 | 0.456697 | 3545 |
| mfqtotal18 | Beta | -0.06878 | -0.25215 | 0.11458 | 0.462086 | 3212 |
| bmi84 | Beta | -0.02001 | -0.07337 | 0.033338 | 0.462135 | 5799 |
| emotion_stable13 | Beta | -0.0679 | -0.25762 | 0.121814 | 0.4829 | 4224 |
| caff_8_MS | Beta | 0.291497 | -0.52996 | 1.112953 | 0.486661 | 4589 |
| cisrphobia18 | Beta | 0.007394 | -0.0142 | 0.02899 | 0.502118 | 3293 |
| sdqtotdif7 | Beta | 0.036584 | -0.08639 | 0.15956 | 0.559791 | 5455 |
| emotional7 | Beta | 0.012371 | -0.02967 | 0.054409 | 0.564045 | 5462 |
| totalcigs23 | Beta | 0.009067 | -0.02561 | 0.043741 | 0.608231 | 7841 |
| eversmoked18 | OR | 0.978499 | 0.894276 | 1.070655 | 0.610646 | 2402 |
| bmi204 | Beta | -0.03084 | -0.16031 | 0.098623 | 0.640453 | 3606 |
| intellect13 | Beta | -0.04001 | -0.20998 | 0.129951 | 0.644437 | 4263 |
| norm_YPC1381 | Beta | -0.00118 | -0.00621 | 0.003844 | 0.645024 | 7841 |
| sh_si | OR | 0.969681 | 0.826783 | 1.137276 | 0.683328 | 3263 |
| specphobia10 | OR | 1.06184 | 0.769238 | 1.465743 | 0.694124 | 5473 |
| IQ_8 | Beta | 0.080009 | -0.34876 | 0.508777 | 0.714515 | 5290 |
| eversmoked23 | OR | 0.985667 | 0.90451 | 1.074106 | 0.722464 | 2792 |
| Gr_ODD_7 | Beta | 0.003604 | -0.01679 | 0.023999 | 0.729061 | 4859 |
| autism | OR | 0.955369 | 0.706855 | 1.291254 | 0.748575 | 5381 |
| Gr_lifeevents_7 | Beta | 0.002778 | -0.01525 | 0.02081 | 0.762637 | 5496 |
| dailyalcamt18 | Beta | 0.007029 | -0.03863 | 0.052682 | 0.762771 | 2826 |
| sleep15_maint | Beta | 0.003845 | -0.02143 | 0.02912 | 0.765513 | 3419 |
| cisrdep5sum | Beta | 0.019277 | -0.11044 | 0.148997 | 0.770792 | 3303 |
| fh5440_16 | Beta | 0.004487 | -0.02673 | 0.035706 | 0.778118 | 3727 |
| sdqtotdif17 | Beta | -0.02047 | -0.16953 | 0.128597 | 0.787792 | 4055 |
| Gr_CD_7 | Beta | 0.002406 | -0.01607 | 0.020887 | 0.798556 | 5329 |
| agreeable13 | Beta | -0.01762 | -0.16208 | 0.126846 | 0.811054 | 4279 |
| Gr_lifeevents_16 | Beta | 0.002178 | -0.01603 | 0.020385 | 0.814589 | 3378 |
| mfqtotal14 | Beta | 0.013435 | -0.11549 | 0.142364 | 0.838139 | 4574 |
| sleep15_init | Beta | -0.04927 | -0.53194 | 0.433388 | 0.841371 | 3627 |
| smoked14 | OR | 0.989377 | 0.880706 | 1.111457 | 0.845877 | 2089 |
| age1stsmoked | Beta | 0.004026 | -0.03821 | 0.046262 | 0.851647 | 1064 |
| tipsyamt18 | Beta | -0.01149 | -0.13544 | 0.112461 | 0.855797 | 2391 |
| IQ_15 | Beta | -0.03816 | -0.45692 | 0.380596 | 0.858204 | 3721 |
| mfqtotal10 | Beta | 0.006555 | -0.07908 | 0.092188 | 0.880719 | 5434 |
| anxiety8 | Beta | 0.003345 | -0.0433 | 0.049996 | 0.888199 | 5358 |
| ptsd15 | Beta | -0.00082 | -0.01455 | 0.0129 | 0.906301 | 4009 |
| sleep7_init_bi | OR | 0.996684 | 0.937443 | 1.059669 | 0.908574 | 5479 |
| psychosis12_pos | Beta | -0.00056 | -0.0143 | 0.013185 | 0.936597 | 4974 |
| gcse_AC | OR | 0.994563 | 0.775584 | 1.275369 | 0.962949 | 2360 |
| emotional17 | Beta | 0.001033 | -0.05589 | 0.057952 | 0.971617 | 4073 |
| psychosis18_pos | Beta | -0.00011 | -0.01597 | 0.015747 | 0.989022 | 3403 |

| **Supplementary Table 12: Associations between maternal alcohol PRS and offspring mental health phenotype (intergenerational) excluding ALSPAC and 23andMe from summary statistics used to make PRS** | | | | | | |
| --- | --- | --- | --- | --- | --- | --- |
| Phenotype | Type | OR/beta | lowerCI | upperCI | pvalue | n |
| Intellectual ability | beta | -0.2198 | -0.39146 | -0.04815 | 0.012095 | 3956 |
| AUDIT total score age 24 | beta | 0.181373 | 0.017657 | 0.345088 | 0.029919 | 2516 |
| Exercise frequency | beta | 0.026152 | 0.001186 | 0.051118 | 0.040067 | 3969 |
| Lifetime cigarettes smoked age 14 | OR | 1.259356 | 0.984525 | 1.610905 | 0.064079 | 421 |
| Daily caffeine intake age 8 | beta | 0.768465 | -0.05244 | 1.589368 | 0.066533 | 4067 |
| GCSE D-G | OR | 0.919645 | 0.833199 | 1.015059 | 0.089756 | 2038 |
| BMI age 7 | beta | -0.04642 | -0.10221 | 0.009365 | 0.102878 | 5032 |
| Conscientiousness | beta | -0.14865 | -0.33085 | 0.033556 | 0.10979 | 3863 |
| Number of times had whole alcoholic drink | beta | 0.407849 | -0.0952 | 0.910899 | 0.111931 | 1012 |
| Depression age 10 | beta | 0.071006 | -0.01988 | 0.161889 | 0.125664 | 4885 |
| Education age 15 | beta | 0.318591 | -0.12474 | 0.761918 | 0.158927 | 3450 |
| Ever smoked age 23 | OR | 1.060008 | 0.97205 | 1.155925 | 0.169826 | 2610 |
| BMI age 17 | beta | -0.09386 | -0.23142 | 0.043696 | 0.181031 | 3353 |
| Total behavioural difficulties age 7 | beta | 0.072133 | -0.0541 | 0.198371 | 0.262682 | 5135 |
| Age 1st smoked | beta | -0.0238 | -0.06719 | 0.019586 | 0.281926 | 970 |
| Psychosis positive symptoms age 18 | beta | -0.00844 | -0.02454 | 0.007657 | 0.303974 | 3180 |
| Depression age 14 | beta | 0.068149 | -0.06597 | 0.202272 | 0.319233 | 4250 |
| Lifetime cigarettes smoked age 23 | beta | 0.016505 | -0.01912 | 0.052125 | 0.363749 | 7727 |
| Depression age 18 | beta | -0.08799 | -0.27829 | 0.1023 | 0.364652 | 3015 |
| Extraversion | beta | 0.096885 | -0.11284 | 0.306615 | 0.36516 | 4046 |
| Conduct disorder age 16 | beta | 0.020332 | -0.02374 | 0.064399 | 0.365731 | 3538 |
| Anxiety symptoms score age 18 | beta | -0.01153 | -0.03734 | 0.01428 | 0.381134 | 3051 |
| Agreeableness | beta | -0.06714 | -0.21886 | 0.08458 | 0.385663 | 3983 |
| Psychosis negative symptoms age 16 | beta | 0.079409 | -0.10253 | 0.261344 | 0.392182 | 3273 |
| Sleep initiation age 15 | beta | -0.22632 | -0.74911 | 0.296478 | 0.396069 | 3355 |
| Lifetime cigarettes smoked age 18 | beta | -0.04148 | -0.13748 | 0.054531 | 0.396788 | 1041 |
| Ever smoked cannabis | OR | 1.035108 | 0.950411 | 1.127352 | 0.398378 | 3335 |
| Hyperactivity symptoms | beta | 0.007513 | -0.01032 | 0.025342 | 0.408802 | 4918 |
| Emotional symptoms score age 17 | beta | 0.025284 | -0.03479 | 0.085362 | 0.409354 | 3765 |
| Binge drinking age 18 | beta | 0.019667 | -0.02735 | 0.06668 | 0.412121 | 2651 |
| Ever smoked age 18 | OR | 1.035431 | 0.944199 | 1.135479 | 0.42947 | 2239 |
| Smoked age 14 | OR | 1.045343 | 0.927927 | 1.177616 | 0.435821 | 1950 |
| GCSE A-C | OR | 1.08562 | 0.864017 | 1.364059 | 0.450875 | 2204 |
| AUDIT total score age 18 | beta | 0.066028 | -0.10655 | 0.23861 | 0.453206 | 2812 |
| Sleep maintenance age 7 | OR | 0.977091 | 0.91509 | 1.043293 | 0.458678 | 5129 |
| Conduct disorder age 7 | beta | 0.007227 | -0.01251 | 0.026961 | 0.472826 | 5014 |
| Alcohol frequency age 18 | beta | 0.011104 | -0.02026 | 0.042468 | 0.487629 | 2702 |
| Number of cigarettes smoked daily age 23 | beta | 0.002145 | -0.00398 | 0.008266 | 0.492252 | 7727 |
| Average drink total per day age 18 | beta | -0.01437 | -0.06007 | 0.031334 | 0.5376 | 2647 |
| Total behavioural difficulties age 17 | beta | 0.049893 | -0.1102 | 0.209982 | 0.541213 | 3748 |
| Life events age 16 | beta | -0.00599 | -0.02522 | 0.013254 | 0.541908 | 3148 |
| Sleep maintenance age 15 | beta | 0.00821 | -0.0182 | 0.034618 | 0.54218 | 3179 |
| Education age 8 | beta | 0.142337 | -0.32673 | 0.611406 | 0.551939 | 4675 |
| Phobia symptom score | beta | 0.006871 | -0.01601 | 0.02975 | 0.556006 | 3051 |
| Anxiety age 8 | beta | 0.013978 | -0.03353 | 0.061489 | 0.56411 | 4995 |
| AUDIT risk score age 18 | beta | 0.006689 | -0.01715 | 0.030526 | 0.582184 | 2812 |
| Frequency smokes cannabis | beta | -0.02026 | -0.09505 | 0.054527 | 0.595078 | 960 |
| Sleep initiation age 7 | OR | 1.015693 | 0.954373 | 1.080953 | 0.598195 | 5152 |
| Emotional stability | beta | 0.053192 | -0.15128 | 0.257661 | 0.610054 | 3901 |
| Suicide attempt | OR | 1.035159 | 0.880153 | 1.217462 | 0.652976 | 3076 |
| Sleep duration age 16 | beta | -0.00705 | -0.03919 | 0.02508 | 0.6669 | 3456 |
| Eating disorder 16 | OR | 1.053194 | 0.78246 | 1.417603 | 0.71239 | 3310 |
| Emotional symptoms score age 7 | beta | 0.008537 | -0.03698 | 0.054055 | 0.713118 | 5141 |
| Eating disorder 13 | OR | 1.08535 | 0.648787 | 1.815672 | 0.736443 | 3956 |
| Autism | OR | 1.049578 | 0.750726 | 1.467396 | 0.760024 | 4910 |
| PTSD | beta | 0.002036 | -0.01232 | 0.016392 | 0.780983 | 3722 |
| Sleep duration | beta | -0.00308 | -0.02593 | 0.019767 | 0.791598 | 5129 |
| Oppositional defiant disorder age 15 | beta | 0.00354 | -0.02311 | 0.030194 | 0.794574 | 2759 |
| Handedness | OR | 1.010565 | 0.920044 | 1.109991 | 0.812604 | 4849 |
| Depression age 18 | beta | 0.015603 | -0.1235 | 0.154711 | 0.825941 | 3059 |
| ADHD | beta | 0.004055 | -0.03303 | 0.041144 | 0.830259 | 3566 |
| Specific phobia | OR | 0.974026 | 0.745334 | 1.272888 | 0.835047 | 5102 |
| Number of drinks to feel tipsy | beta | -0.0113 | -0.12793 | 0.105324 | 0.849295 | 2246 |
| Smoked age 18 | beta | -0.00994 | -0.14223 | 0.122346 | 0.882798 | 1038 |
| Psychosis positive symptoms age 12 | beta | -0.00074 | -0.01571 | 0.014238 | 0.92313 | 4568 |
| Oppositional defiant disorder age 7 | beta | 0.000385 | -0.02086 | 0.021631 | 0.971646 | 4492 |
| Life events age 7 | beta | -0.00027 | -0.01885 | 0.018303 | 0.976956 | 5169 |
| Number of cigarettes smoked weekly age 23 | beta | 3.86E-05 | -0.00478 | 0.004854 | 0.987457 | 7727 |
| Intellectual ability | beta | -0.2198 | -0.39146 | -0.04815 | 0.012095 | 3956 |
| AUDIT total score age 24 | beta | 0.181373 | 0.017657 | 0.345088 | 0.029919 | 2516 |
| Exercise frequency | beta | 0.026152 | 0.001186 | 0.051118 | 0.040067 | 3969 |
| Lifetime cigarettes smoked age 14 | OR | 1.259356 | 0.984525 | 1.610905 | 0.064079 | 421 |
| Daily caffeine intake age 8 | beta | 0.768465 | -0.05244 | 1.589368 | 0.066533 | 4067 |
| GCSE D-G | OR | 0.919645 | 0.833199 | 1.015059 | 0.089756 | 2038 |
| BMI age 7 | beta | -0.04642 | -0.10221 | 0.009365 | 0.102878 | 5032 |
| Conscientiousness | beta | -0.14865 | -0.33085 | 0.033556 | 0.10979 | 3863 |
| Number of times had whole alcoholic drink | beta | 0.407849 | -0.0952 | 0.910899 | 0.111931 | 1012 |
| Depression age 10 | beta | 0.071006 | -0.01988 | 0.161889 | 0.125664 | 4885 |
| Education age 15 | beta | 0.318591 | -0.12474 | 0.761918 | 0.158927 | 3450 |
| Ever smoked age 23 | OR | 1.060008 | 0.97205 | 1.155925 | 0.169826 | 2610 |
| BMI age 17 | beta | -0.09386 | -0.23142 | 0.043696 | 0.181031 | 3353 |
| Total behavioural difficulties age 7 | beta | 0.072133 | -0.0541 | 0.198371 | 0.262682 | 5135 |
| Age 1st smoked | beta | -0.0238 | -0.06719 | 0.019586 | 0.281926 | 970 |
| Psychosis positive symptoms age 18 | beta | -0.00844 | -0.02454 | 0.007657 | 0.303974 | 3180 |

***Supplementary Methods:***

*Cohort sample:* Pregnant women resident in Avon, UK with expected dates of delivery 1st April 1991 to 31st December 1992 were invited to take part in the study. The initial number of pregnancies enrolled is 14,541 (for these at least one questionnaire has been returned or a “Children in Focus” clinic had been attended by 19/07/99). Of these initial pregnancies, there was a total of 14,676 fetuses, resulting in 14,062 live births and 13,988 children who were alive at 1 year of age. When the oldest children were approximately 7 years of age, an attempt was made to bolster the initial sample with eligible cases who had failed to join the study originally. As a result, when considering variables collected from the age of seven onwards (and potentially abstracted from obstetric notes) there are data available for more than the 14,541 pregnancies mentioned above. The number of new pregnancies not in the initial sample (known as Phase I enrolment) that are currently represented on the built files and reflecting enrolment status at the age of 24 is 913 (456, 262 and 195 recruited during Phases II, III and IV respectively), resulting in an additional 913 children being enrolled. The phases of enrolment are described in more detail in the cohort profile paper and its update (Boyd et al., 2013; Fraser et al., 2013; Northstone et al., 2019). The total sample size for analyses using any data collected after the age of seven is therefore 15,454 pregnancies, resulting in 15,589 fetuses. Of these 14,901 were alive at 1 year of age. A 10% sample of the ALSPAC cohort, known as the Children in Focus (CiF) group, attended clinics at the University of Bristol at various time intervals between 4 to 61 months of age. The CiF group were chosen at random from the last 6 months of ALSPAC births (1432 families attended at least one clinic). Excluded were those mothers who had moved out of the area or were lost to follow-up, and those partaking in another study of infant development in Avon. Please note that the study website contains details of all the data that is available through a fully searchable data dictionary and variable search tool: <http://www.bristol.ac.uk/alspac/researchers/our-data/>.

*Alcohol PRS:* Due to multiple cohorts included within their analyses, varying measures of alcohol consumption were recorded. If included studies used categorical responses (e.g., 1-5 drinks per week) the midpoint was used (e.g., 1-5 drinks per week was recorded as 2.5 drinks per week). To stop potential outliers driving results, the drinks per week phenotype was left-anchored at 1 and log-transformed by Liu and colleagues prior to analyses.

*Phenotyping:* Where multiple measures were available which measured the same construct or the same underlying phenotype at the same age, each were tested for correlation. If variables were found to be highly correlated, the variable with the biggest sample size was selected to maximise power. Continuous variables were favoured over binary variables of the same measure. All continuous phenotypes were checked for zero-inflated variables and normal distribution, and those that were not normally distributed were transformed into quantiles of categorical traits. Phenotypes with >20% zero-inflation were rank transformed into 3 quantiles (0, ≤ median, > median). Non-ordered categorical phenotypes were transformed into binary variables where appropriate, indicating the presence of an outcome compared to a control. All non-binary variables were treated as continuous in linear regression analyses.

*Genotyping:* Children from the ALSPAC cohort were genotyped using the Illumina HumanHap550 quad chip genotyping platforms by 23andme subcontracting the Wellcome Trust Sanger Institute, Cambridge, UK and the Laboratory Corporation of America, Burlington, NC, US. Mothers from ALSPAC were genotyped using the Illumina human660W-quad array at Centre National de G*énotypage* (CNG) and genotypes were called with Illumina GenomeStudio. PLINK (v1.07) was used to carry out quality control measures on an initial set of 10,015 subjects and 557,124 directly genotyped SNPs. Mothers SNPs were removed if they displayed more than 5% missingness or a Hardy-Weinberg equilibrium P value of <1.0e-06. Population stratification was assessed by multidimensional scaling analysis and compared with Hapmap II (release 22) European descent (CEU), Han Chinese, Japanese and Yoruba reference populations; all individuals with non-European ancestry were removed. This then combined 477,482 SNP genotypes in common between the sample of mothers and sample of children. Additionally, if any SNPs had a minor allele frequency of <1% or had genotype missingness >1% because of poor genotyping, they were also removed (11,396 SNPs removed). A further 321 subjects were removed because of potential ID mismatches. After this genotyping and quality control, a dataset of 17,842 subjects remained. This contained 6,305 duos and 465,740 SNPs. After related subjects were excluded, 8,237 eligible children and 8,196 eligible mothers with available genotype data remained. Further detail of the quality control process is described in (Paternoster et al., 2011, Taylor et al., 2018).

*Phenotyping*

### *Mother phenotypes*

All maternal phenotypes were self-reported. Measures of maternal alcohol use during pregnancy were continuous measures of, alcohol consumption in months 1-3 of pregnancy, number of days mothers drank 4+ units of alcohol in the past month (binge drinking) at 18 and 32 weeks gestation, total weekly units of alcohol consumed at 32 weeks gestation. Binary measures were, the most consumed alcoholic beverage (compared to other alcoholic drinks during pregnancy) at 18 weeks gestation, being wine, beer or lager, spirits, sherry or port, other types of alcohol. As well as measures indicating change in alcohol consumption during pregnancy or not (increased use/stopped use/no change/increased craving/never used), for mothers who normally consumed alcohol and those who normally did not consume alcoholic drinks. An overview of included phenotypes can be seen in Fig. 1.

Measures of maternal mental health and other substance use collected at 8 weeks gestation were: changes (increased use/stopped use/no change/increased craving/never used) to caffeine and tobacco consumption. Collected at 18 weeks gestation were: neurotic symptomatology (Crown Crisp Experiential Index), hypersensitivity to personal rejection, image perception within the last four weeks, image perception change from 3 months pre-pregnancy to 18 weeks gestation, reactions towards becoming a parent, illicit drug use, smoked during pregnancy, taken cannabis during pregnancy, vomited during pregnancy and if mothers had ever smoked in their lifetime. Collected at 32 weeks gestation were: total caffeine consumption, highest maternal education qualification, mother’s perception of their own physical activity compared to other pregnant women of a similar age. Depression (Edinburgh Postnatal Depression Scale (EPDS) (Cox, Chapman, Murray, & Jones, 1996)) was measured at both 18 and 32 weeks gestation, with scores ≥13 indicating depression diagnosis.

Demographic measures included were: maternal socioeconomic status, adverse life events (asked during pregnancy at 32 weeks gestation), and a combined measure of average income (measured at 33 and 47 months).

### *Child phenotypes*

Offspring phenotypes were included as close to age 18 as possible; further analyses using the same phenotypes (where available) as close to age 7 as possible were included as negative controls. Measures of adolescent alcohol use self-reported at age 18 were: alcohol frequency, amount of alcohol consumed daily, number of days they drank 4+ units of alcohol in the past month, number of drinks it took to feel tipsy, total score for hazardous drinking behaviours from the Alcohol Use Disorders Identification Test (AUDIT) and number of times they had consumed alcohol. Total AUDIT score was also included at age 24. An overview of child and adolescent phenotypes included can be seen in Fig. 2.

Adolescent substance use measures were: caffeine intake, ever tried cannabis, frequency of cannabis use, ever smoked tobacco, age first smoked tobacco, total number of cigarettes smoked in lifetime and number of cigarettes smoked per day and per week.

Adolescent mental health measures included were: personality (agreeableness, extraversion, emotional stability, intellectual ability and conscientiousness), phobia symptoms, total anxiety score, depression (Clinical Interview Schedule-Revised (CIS-R) (Lewis, Pelosi, Araya, & Dunn, 1992) and Short Mood and Feelings Questionnaire (SFMQ) (Angold et al., 1995)), psychotic like symptoms (PLIKS), negative psychotic symptom scores (Community Assessment of Psychotic Experiences (CAPE) (Yung et al., 2009)), total emotional symptoms, total problem score, total attention deficit hyperactive disorder (ADHD) score, conduct disorder (Strengths and Difficulties Questionnaire (SDQ) (Goodman, 1997; Woerner et al., 2004)), oppositional defiant disorder (ODD), post-traumatic stress disorder (Development and Wellbeing Assessments (DAWBA)), initiating and difficulty maintaining sleep, average sleep duration on a school night, diagnosis of autism, presence of an eating disorder, and ever self-harmed with suicidal intent.

Adolescent demographic measures included were: body mass index (BMI), frequency of exercise, negative life events, IQ and if obtained a GCSE grade at levels A*-C, or D-G.

Measures collected around age 7 were: number of hours the child normally sleeps during term-time, difficulty initiating and maintaining sleep, presence of ODD, conduct disorder symptoms, hyperactivity symptoms, emotional symptoms score, and total behavioural difficulties, general anxiety symptoms scale, child’s IQ and daily caffeine intake, total depression score and clinical diagnosis of specific phobias.

Child demographic measures included BMI and number of negative life events experienced at age 7. A measure of handedness was included as a further negative control which we did not expect to be associated with the PRS regardless of own alcohol exposure.

*Bonferroni correction*

Phenotypes were categorised by types of variable into 4 categories, alcohol, other substances (tobacco, caffeine, other substances), internalising problems, externalising problems and demographic and mental health related variables. The number of tests used within each of these 3 categories was then used within Bonferroni correction separately for analyses of maternal alcohol PRS and maternal outcomes, child alcohol PRS and child outcomes and maternal alcohol PRS and child outcomes. For Bonferroni correction thresholds we used an α value of 0.05 / number of tests within each analysis: alcohol (maternal: 15 tests, *p =* 0.003; offspring: 7 tests, *p =* 0.007), other substances (maternal: 15 tests, *p =* 0.003; offspring: 12 tests, *p =* 0.004), internalising problems (maternal: 6 tests, *p =* 0.008; offspring: 17 tests, *p* = 0.003), externalising (maternal: 0 tests; offspring: 8 tests, *p* = 0.006), other mental health related variables (maternal: 7 tests, *p =* 0.007; offspring: 24 tests, *p* = 0.002).
